# Correlation Analysis of Gut Microbiota and Serum Metabolome With *Porphyromonas gingivalis*-Induced Metabolic Disorders

**DOI:** 10.3389/fcimb.2022.858902

**Published:** 2022-04-07

**Authors:** ZhengJie Dong, WanQi Lv, ChenYang Zhang, Si Chen

**Affiliations:** ^1^ Department of Implantology, Shanghai Stomatological Hospital and School of Stomatology, Fudan University, Shanghai, China; ^2^ Shanghai Key Laboratory of Craniomaxillofacial Development and Diseases, Fudan University, Shanghai, China; ^3^ State Key Laboratory of Molecular Engineering of Ploymers, Fudan University, Shanghai, China

**Keywords:** *Porphyromonas gingivalis*, metabolic disorders, intestinal permeability, gut microbiota, serum metabolome, correlation analysis

## Abstract

Periodontitis has been demonstrated to increase the risk of metabolic syndrome (MetS), but the underlying mechanism remains unclear. Recent studies have indicated periodontopathic bacteria such as *Porphyromonas gingivalis* could induce gut microbiota (GM) dysbiosis and aggravate metabolic disorders. However, the effects of microbial metabolites have barely been evaluated. Here, we investigated the alteration of serum metabolome with *P. gingivalis*-induced metabolic disorders, and explored the correlations of GM and serum metabolites. In this study, we orally administered *P. gingivalis* ATCC33277 to C57BL/6 mice and performed metagenomic sequencing and untargeted metabolomics with fecal samples and serum collection. *In vivo* experiments showed a higher proportion of fat mass and worse glucose tolerance in *P. gingivalis*-administered mice, accompanied with an increase of adipose inflammation and gut permeability, which was similar to HFD-induced obese mice. Metagenomic sequencing indicated a compositional and functional alteration of GM. Untargeted metabolomics revealed an alteration of metabolites in *P. gingivalis*-administered mice, and most of them were engaged in metabolic pathways, such as tryptophan metabolism and choline metabolism. Correlation analysis between GM and serum metabolome indicated strong relativity with *P. gingivalis* administration. These results demonstrated some specific microbiota-derived metabolites in the pathogenesis of *P. gingivalis*-induced metabolic disorders, providing promising targets for the development of novel treatment strategies for MetS.

## Introduction

With the global epidemic of obesity and diabetes, the incidence of metabolic disorders has skyrocketed, which will bring huge social and economic burdens (Miranda et al., 2005). Metabolic syndrome (MetS), also known as insulin resistance syndrome, refers to a cluster of physical risk factors (including abdominal obesity; elevated triglycerides, fasting glucose, and blood pressure; and reduced high-density lipoprotein cholesterol) that are associated with increased risks of cardiovascular disease (Broekhuizen et al., 2011; Zomer et al., 2014; Khalil et al., 2018), T2D (Wannamethee et al., 2005; Rohm et al., 2022) and even mortality (Kassi et al., 2011; He et al, 2021). Accumulating evidence has recognized MetS to be a systemic proinflammatory state with gut flora dysbiosis and adipose inflammation as putative pathogenic mechanisms. A series of studies showed that chronic high-fat diets (HFDs) could alter gut microbiota composition and cause defects of the intestinal barrier that facilitate enterotoxin entry into systemic circulation (Cani et al., 2007; Greenhill, 2015; Alveirinho et al., 2020). Meanwhile, rapid hypertrophy of adipocytes results in tissue hypoxia and secretion of adipokines (such as IL-6 and TNF-α), which recruit macrophages to secrete proinflammatory factors, exacerbating systemic inflammation and further contributing to insulin resistance (Lumeng et al, 2007; Winer et al., 2016).

Periodontitis, a chronic inflammatory disease of the tooth-supporting apparatus, has recently been linked to metabolic syndrome due to its common denominator of inflammation and its close correlation with systemic diseases (Watanabe and Cho, 2014; Pirih et al., 2021). Patients with severe periodontitis have a higher risk for MetS (Saito et al, 2005; Lamster and Pagan, 2017; Almoznino et al., 2021), and the periodontal probing depth is positively correlated with the prevalence of MetS (Francesco et al., 2008; Kim et al., 2018). Moreover, another investigation showed that the frequency of brushing teeth was inversely proportional to the incidence of metabolic syndrome, and people who brushed their teeth ≥ 3 times a day had a lower risk of diabetes, dyslipidemia and metabolic syndrome (Kobayashi et al., 2012).

As a biofilm-mediated dysbiosis, periodontitis is predominantly initiated with anaerobic gram-negative bacteria, among which *Porphyromonas gingivalis* has been most elucidated (Smyth, 2011; Lamont et al., 2018). Given that the oral mucosa and colonic mucosa are physically connected, oral bacteria could be ingested and translocated to the lower digestive tract, and a possible mechanism of the “mouth-gut axis” has been proposed in the pathogenesis of periodontitis-associated MetS. Notably, a few studies conducted recently demonstrated that oral administration of periodontal pathogens, mainly *P. gingivalis*, could cause a variety of MetS-related disorders, including increased BMI, insulin resistance, circulatory inflammatory states, and alterations of intestinal permeability and microbiota (Blasco-Baque et al., 2017; Tsuzuno et al., 2021; Yamazaki et al., 2021). However, little research has focused on the alteration of gut microbiota-derived metabolites. As byproducts of gut microbiota, metabolites are key factors in host-microbiota crosstalk. Specific classes of metabolites, notably short-chain fatty acids (SCFAs), bile acids, trimethylamine N-oxide (TMAO), and tryptophan and indole derivatives, have been strongly implicated in the pathogenesis of MetS.

## Methods

### Cultivation of *P. gingivalis*

*P. gingivalis* strain ATCC33277 was obtained from ATCC, and cultured in a brain–heart infusion (BD Bioscience, Franklin Lakes, NJ) containing 0.5% yeast extract (BD Bioscience), 10 mg/L hemin (Wako Chemicals, Osaka, Japan) and 1 mg/L 2-methyl-1,4-naphthoquinone (vitamin K3) (Tokyokasei, Tokyo, Japan) and incubated under anaerobic conditions (80% N_2_, 10% CO_2_, and 10% H_2_) at 37°C. Bacterial suspensions were prepared in phosphate-buffered saline (PBS) without Mg^2+^/Ca^2+^, and the optical density (OD) was measured at 600 nm with a standard curve.

### Animal Experiments

All animal experiments were approved by the Committee for the Care and Use of Laboratory Animals at Fudan University. C57BL/6 male mice (8 weeks old, Beijing Vital River Laboratory Animal Technology Co., Ltd., Beijing, China) were group-housed in a specific pathogen-free (SPF) controlled environment with free access to food and water under a strict 12 h light/dark cycle. Forty mice were randomized into four equal groups with ten mice each: ND (normal diet), HFD (high-fat diet), sham, and *Pg*.

For the obese model, mice were fed a high-fat diet (HFD, 60% fat, 20% protein, and 20% carbohydrates, Research Diets, D12492) to induce obesity for 12 weeks, while a normal diet (ND, 10% fat, 20% protein, and 70% carbohydrates, Research Diets, D12450J) was used as a control. For *P. gingivalis* administration, the mice were gavaged with 10^9^ CFU *P. gingivalis* twice a week for 6 weeks, and PBS with 2% carboxymethylcellulose was administered as a sham. Body weight was assessed in the last week, and fat mass was detected using a Minispec LF90 body composition analyzer (Bruker, Massachusetts, USA).

### Intraperitoneal Glucose Tolerance Test

Mice fasting for 6 h were injected with glucose (1 g/kg) intraperitoneally. Blood glucose was measured with tail vein blood at 0 min, 15 min, 30 min, 60 min, 90 min and 120 min using a OneTouch Ultra glucometer (LifeScan, Pennsylvania, USA). The area under the curve (AUC) of the glucose level over time was calculated to evaluate the glucose tolerance ability.

### HOMA-IR

The concentration of serum insulin was determined by an ELISA kit (Solarbio, Beijing, China) according to the manufacturer’s instructions. HOMA-IR in mice was then calculated using the equation (fasting glucose concentration * fasting insulin concentration)/405.

### Quantitative Real-Time PCR

Total RNA was extracted from the subcutaneous white adipose tissue with TRIzol^®^ reagent (Invitrogen, California, USA). Reverse transcription was conducted with a SuperScript First-Strand cDNA Synthesis kit (Invitrogen) according to the manufacturer’s instructions. Quantitative real-time PCR (qRT–PCR) was performed with SYBR Green Master Mix (Roche Applied Science, Mannheim, Germany). Gene expression was detected with a 7500 real-time PCR system (Applied Bioscience, Foster City, CA, USA), and the thermal settings used were as follows: 95°C for 10 min, followed by 40 cycles of 95°C for 15 s and 65°C for 1 min. The primers (Shanghai Life Biotechnology Co., Ltd., Guangzhou, China) used are listed as following: *TNF-α*, forward primer: 5’- TGCCTATGTCTCAGCCTCTTC-3’, reverse primer: 5’- GGTCTGGGCCATAGAACTGA-3’; *Cd68*, forward primer: 5’-TGTCTGATCTTGCTAGGACCG-3’, reverse primer: 5’-GAGAGTAACGGCCTTTTTGTGA-3’; *Adgre1*, forward primer: 5’-TGACTCACCTTGTGGTCCTAA-3’, reverse primer: 5’-CTTCCCAGAATCCAGTCTTTCC-3’; *Adipoq*, forward primer: 5’-TGTTCCTCTTAATCCTGCCCA-3’, reverse primer: 5’-CCAACCTGCACAAGTTCCCTT-3’. *Gapdh* was utilized for normalization. Data were analyzed using the 2^-ΔΔCt^ relative expression method.

### HE Staining

Subcutaneous white adipose tissue in all four groups and colon samples from the *Pg* and sham groups were harvested immediately after euthanization. Tissues were fixed in adipose tissue fixative or 10% formalin, embedded in paraffin, cut into 5 μm-thick sections and stained with hematoxylin and eosin (HE).

### Immunofluorescence Assay

For immunofluorescence staining, sections were deparaffinized, rehydrated and treated with 3% H_2_O_2_ in methanol for 20 min to inactivate endogenous peroxidase activity. Antigen retrieval was conducted with 1% pepsin (Sigma–Aldrich, St. Louis, USA) and treated with serum albumin (BSA) for 1 h. For adipose tissues, sections were incubated with anti-F4/80 antibody (1:100, Abcam, Cambridge, MA, USA) and anti-Plin1 antibody (1:100, Abcam) overnight at 4°C, followed by three washes (5 min) in PBS. For colon tissues, sections were incubated with anti-ZO-1 antibody (1:100; Proteintech, Wuhan, China) and anti-E-cadherin antibody (1:100; Proteintech) overnight at 4°C. The secondary antibody anti-rabbit (Abcam) was applied for 1 hour at room temperature in the dark, followed by three washes (5 min) in PBS. Slides were then mounted with Vectashield hardset mounting media containing 4’,6-diamidino-2-phenylindole (DAPI). Images were obtained using an Olympus BX51 (Olympus America, Melville, NY) epifluorescence microscope, and images were captured with an Olympus DP-70 camera.

### Intestinal Permeability Assay

Intestinal permeability was determined by FITC-dextran assay. Briefly, mice fasting for 6 hours were orally administered 150 μL FITC-conjugated dextran (FITC-dextran; Sigma–Aldrich) at 80 mg/mL. Four hours later, blood was centrifuged at 1,000 x g for 30 min at 4°C for serum collection. The concentration of fluorescein was quantified at an excitation wavelength of 485 nm and an emission wavelength of 535 nm, and serially diluted samples of the FITC-dextran marker were used as standards.

### Metagenomics Analysis

Total genomic DNA was extracted from the colon contents of mice in the *Pg* and sham groups using a PowerSiol^®^ DNA isolation kit (MO BIO, Carlsbad, CA). DNA extraction was fragmented to an average size of approximately 300 bp, and the paired-end library was constructed using NEXTFLEX^®^ Rapid DNA-Seq (Bioo Scientific, Austin, TX, USA). Paired-end sequencing was performed on an Illumina NovaSeq/HiSeq Xten (Illumina Inc., San Diego, CA, USA) using NovaSeq Reagent Kits/HiSeq X Reagent Kits. Reads that were less than 50 bp, had a quality value < 20 or had N bases were removed. Metagenomics data were assembled using MEGAHIT, and contigs with lengths greater than or greater than 300 bp were selected as the final assembly result. Open reading frames (ORFs) from each assembled contig were predicted using MetaGene. The predicted ORFs with lengths greater than or greater than 100 bp were retrieved and translated into amino acid sequences using the NCBI translation table. All predicted genes with 95% sequence identity and 90% coverage were clustered using CD-HIT. The longest sequences from each cluster were selected as representative sequences for nonredundant gene catalog construction. The quality-controlled reads were mapped to the representative sequences with 95% identity using SOAPaligner, and gene abundance in each sample was evaluated. The KEGG annotation was conducted using BLASTP against the Kyoto Encyclopedia of Genes and Genomes database with an e-value cutoff of 1e^-5^.

### Untargeted Metabolomics Profiling

Untargeted metabolomics profiling of serum samples in the *Pg* and sham groups was performed by Majorbio Bio-Pharm Technology Co. Ltd. (Shanghai, China). Chromatographic separation of the metabolites was performed on a Thermo UHPLC system equipped with an ACQUITY UPLC HSS T3 (100 mm × 2.1 mm i.d., 1.8 µm; Waters, Milford,USA). Mass spectrometry (MS) was performed using a Thermo UHPLC-Q Exactive Mass Spectrometer equipped with an electrospray ionization (ESI) source operating in either positive or negative ion mode. Data acquisition was performed with the Data Dependent Acquisition (DDA) mode. The detection was carried out over a mass range of 70-1050 m/z. Raw data were imported into Progenesis QI 2.3 for peak detection and alignment. The preprocessing results contained the m/z values and peak intensity. The mass spectra of these metabolic features were identified using accurate masses. MS/MS fragment spectra and isotope ratio differences with searches in internal databases and public databases. The variable importance of the projection (VIP) score generated from orthogonal partial least squares discriminate analysis was used to determine the most differentiated metabolites. Metabolites with VIP ≥ 1.0 and *P* value ≤ 0.05 were defined as significantly changed metabolites. A multivariate statistical analysis was performed using the R package ropls version 1.6.2.

### Statistical Analysis

The MetS statistical analysis was conducted with SPSS Statistics 20.0 software (IBM). Data are expressed as the mean ± SD. Differences between two groups were analyzed by Student’s t test. A two-tailed *P* value < 0.05 was considered statistically significant.

## Results

### Assessment of Metabolic Disorders by *P. gingivalis* in Mice

**Figure 1:**
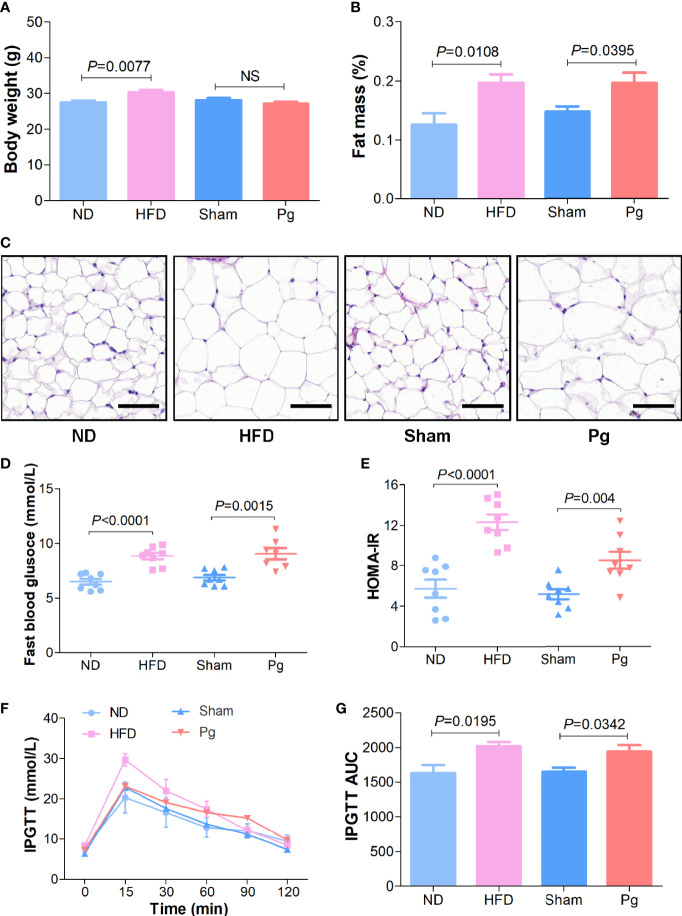
Metabolic syndrome in C57BL/6 mice with *P. gingivalis* administration. **(A)** Body mass of HFD-fed mice and *Pg*-administered mice, with ND or sham as the controls, respectively (n = 6-10 per group). **(B)** Body fat percentage of mice in the ND, HFD, sham and *Pg* groups (n = 6-10 per group). **(C)** Representative images of HE-stained adipose tissues from mice in the ND, HFD, sham and *Pg* groups (Scale bars: 100 μm). **(D)** Fasting blood glucose level of HFD-fed mice and *Pg*-administered mice, with ND or sham as the controls, respectively (n = 8 per group). **(E)** The fasting homeostatic model assessment of insulin resistance (HOMA-IR) of mice in the ND, HFD, sham and *Pg* groups (n = 8 per group). **(F)** Blood glucose level before and after intraperitoneal glucose tolerance test (IPGTT) of HFD-fed mice and *Pg*-administered mice, with ND or sham as the controls, respectively (n = 6-10 per group). **(G)** Area under curve of IPGTT. Differences between two groups were analyzed by a Student’s *t* test. Data are represented as means ± SEM.

To investigate the effects of *P. gingivalis* on MetS, we gavaged C57BL/6 mice with *P. gingivalis* twice a week for 6 weeks, with phosphate-buffered saline (PBS) gavage as the sham group and high-fat diet (HFD)-induced obese mice as the positive control. As the results indicated, HFD increased the body weight compared with the normal diet (ND) group, while no significant difference was observed between the *Pg* group and the sham group (**Figure 1A**). The body composition analysis and HE staining indicated a higher proportion of fat mass and larger adipocyte cells in the HFD and *Pg* groups (**Figures 1B, C**). Moreover, fasting blood glucose was significantly increased in *P. gingivalis*-administered mice, with a similar elevation in the HFD group (**Figure 1D**). The homeostatic model assessment-insulin resistance (HOMA-IR) assessment comprising the fasting blood glucose and insulin level indicated higher insulin resistance in the *Pg* group accompanied by worse glucose tolerance during an intraperitoneal glucose tolerance test (IPGTT) compared with the sham group (**Figures 1E–G**).

### Upregulation of Inflammatory Markers in Adipose Tissue With *P. gingivalis* Administration

**Figure 2:**
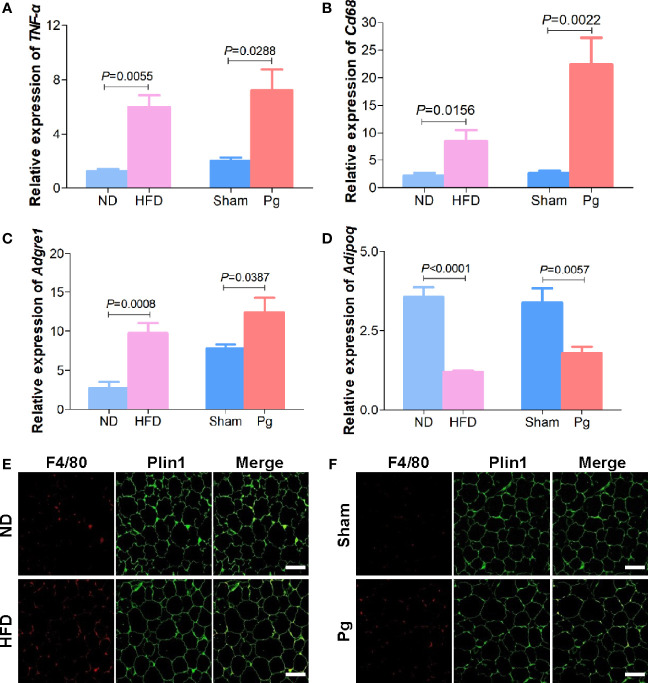
Adipose inflammation in C57BL/6 mice with *P. gingivalis* administration. **(A)** Relative expression of *TNF-α* in the white adipose tissue (WAT) of mice in the ND, HFD, sham and *Pg* groups with qRT-PCR (n = 6-10 per group). **(B, C)** Relative expression of pro-inflammatory factors *Cd68* and *Adgre1* in the WAT of mice in the ND, HFD, sham and *Pg* groups with qRT-PCR (n = 6-10 per group). **(D)** Relative expression of anti-inflammatory factor *Adipoq* in the WAT of mice in the ND, HFD, sham and *Pg* groups with qRT-PCR (n = 6-10 per group). **(E)** Representative images of F4/80 staining in the WAT from mice in the ND and HFD groups. F4/80 was shown in red, and Perilipin1 (Plin1) was shown in green (Scale bars: 100 μm). **(F)** Representative images of F4/80 staining in the WAT from mice in the sham and *Pg* groups. F4/80 was shown in red, and Perilipin1 (Plin1) was shown in green (Scale bars: 100 μm). Differences between two groups were analyzed by a Student’s *t* test. Data are represented as means ± SEM.

Adipose inflammation plays a detrimental role in obesity-related metabolic dysbiosis. Thus, we determined the expression of inflammatory factors in adipose tissue with *P. gingivalis* administration. qRT–PCR indicated the expression of *TNF-α*was increased in the HFD and *Pg* groups(**Figure 2A**). Adipose tissue macrophages play vital roles in obesity-induced inflammation. *Cd68* and *Adgre1* (adhesion G protein-coupled receptor E1), which act as homologs of F4/80, are important markers of macrophages. qRT-PCR demonstrated elevated expression of *Cd68* and *Adgre1* in the HFD and *Pg* groups, with remarkably higher expression of *Cd68* with *P. gingivalis* administration (**Figures 2B, C**). However, the expression of the anti-inflammatory factor *Adipoq* was decreased in the HFD and *Pg* groups (**Figure 2D**). Moreover, immunofluorescence against F4/80 demonstrated higher macrophage infiltration in the adipose tissue of *P. gingivalis*-gavaged mice, similar to the HFD group (**Figures 2E, F**).

### Alteration of Gut Microbiota Composition With *P. gingivalis* Administration

**Figure 3:**
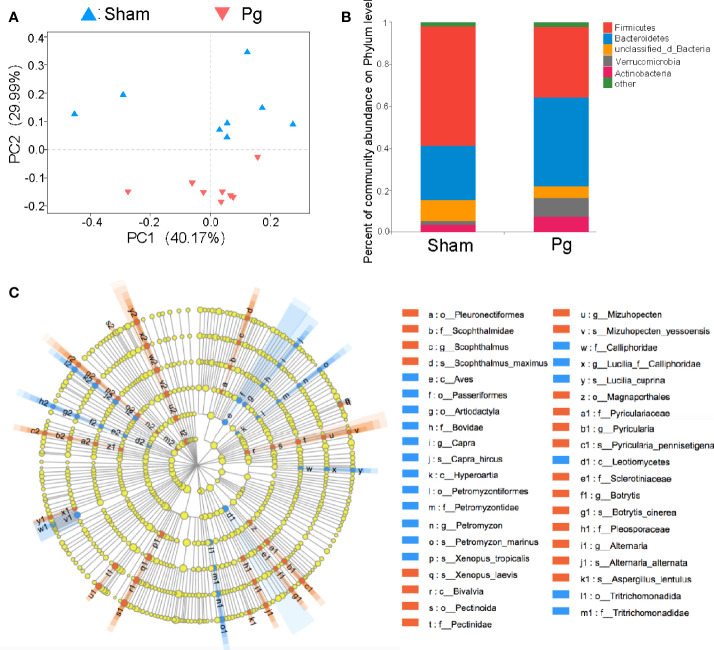
Analysis of gut microbiota in C57BL/6 mice with *P. gingivalis* administration. **(A)** Principal co-ordinates analysis (PCoA) of fecal microbiota from mice in the sham and *Pg* groups using Bray-Curtis distances (n = 8 per group). **(B)** Relative abundances of bacterial groups at the phylum level in the sham and *Pg* groups. **(C)** Linear discriminant analysis effect size (LEfSe) analysis-based cladogram for bacteria in the sham and *Pg* groups based on an LDA score ≥ 3.0.

**Figure 4:**
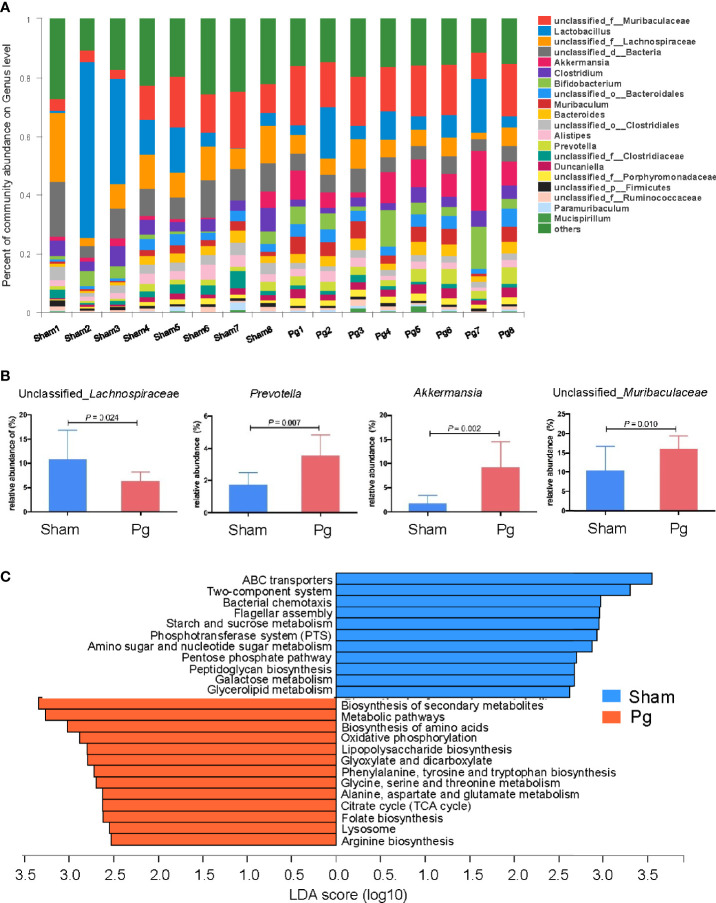
Alteration of gut microbiota at genus level and function with *P. gingivalis* administration. **(A)** Relative abundances of bacterial groups at the genus level in the sham and *Pg* groups (n = 8 per group). **(B)** Bar plots for some genera with significant differences in relative abundance between sham and *Pg* groups. **(C)** Differential KEGG pathways enriched in the sham and *Pg* groups based on an LDA score ≥ 3.0. Non-parametric Kruskal-Wallis sum-rank test was used to detect the significant difference in abundance, and LDA score was used to estimate the impact of the abundance of each component on the differential effect.

Increasing evidence has indicated the role of gut microbiota in the progression of metabolic disorder; thus, we analyzed the composition, abundance and function of gut microbiota with fecal samples in *P. gingivalis*-administered mice *via* metagenome sequencing. The principal coordinate analysis (PCoA) of Bray–Curtis distances revealed significant differences in the composition and abundance of gut microbiota between the *Pg* and sham groups (**Figure 3A**). At the phylum level, the proportion of *Firmicutes* was remarkably lower with *P. gingivalis* administration, while *Bacteroidetes* showed an elevated tendency (**Figure 3B**). The linear discriminant analysis effect size (LEfSe) based on an LDA score ≥ 3.0 demonstrated that the gut microbiota of mice in the *Pg* group was distinguished from that in the sham group by the family, phylum, and genus (**Figure 3C**). At the genus level, the proportion of unclassified *Lachnospiraceae* was decreased in *P. gingivalis*-administered mice, while the proportions of unclassified *Muribaculaceae*, *Akkermansia*, *Prevotella* and *Porphyromonadaceae* were increased (**Figures 4A, B**). Moreover, KEGG annotation showed that mice treated with *P. gingivalis* exhibited a definitely separate enrichment in microbial function and pathways (**Figure 4C**). For example, Biosynthesis of secondary metabolites”, “Metabolic pathways”, and “Biosynthesis of amino acids” were enriched with *P. gingivalis* administration.

### Increased Gut Permeability in *P. gingivalis*-Administered Mice

**Figure 5:**
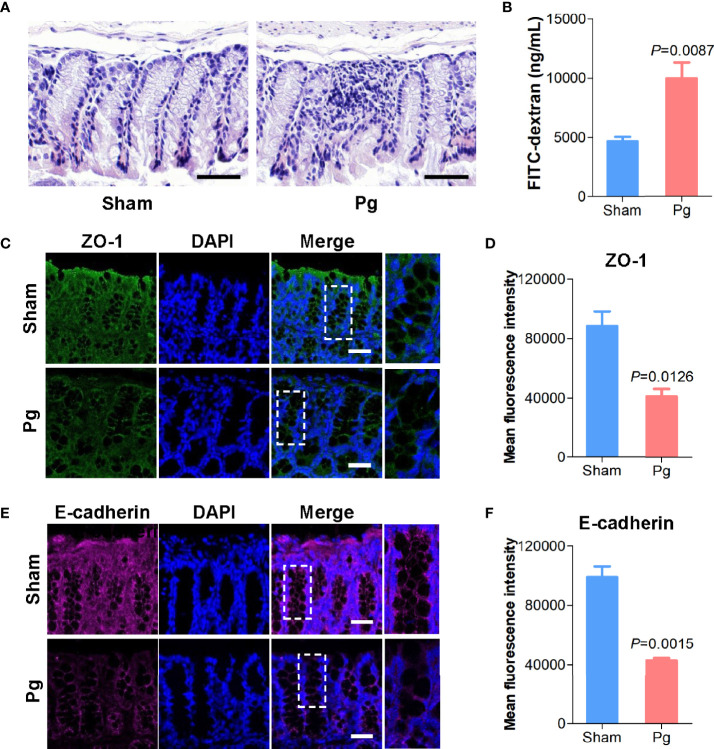
Intestinal permeability in C57BL/6 mice with *P. gingivalis* administration. **(A)** Representative images of HE-stained colon tissues from mice in the sham and *Pg* groups (Scale bars: 100 μm). **(B)** Quantification of serum fluorescein isothiocyanate (FITC)-dextran of mice in the sham and *Pg* groups (n = 6 per group). **(C)** Representative images of ZO-1 staining in the colon sections from mice in the sham and *Pg* groups. ZO-1 was shown in green, and DAPI was shown in blue (Scale bars: 100 μm). **(D)** Quantification of the fluorescence intensity with ZO-1 staining in the sham and *Pg* groups. **(E)** Representative images of E-cadherin staining in the colon sections from mice in the sham and *Pg* groups. E-cadherin was shown in purple, and DAPI was shown in blue (Scale bars: 100 μm). **(F)** Quantification of the fluorescence intensity with E-cadherin staining in the sham and *Pg* groups. Differences between two groups were analyzed by a Student’s *t* test. Data are represented as means ± SEM.

Intestinal barrier function is closely associated with gut microbiota (Dabke et al., 2019); thus, we further investigated gut permeability with *P. gingivalis* administration. HE staining of the intestinal epithelia indicated a higher infiltration of inflammatory cells, such as neutrophils, with *P. gingivalis* administration than in the sham group (**Figure 5A**). The *in vivo* barrier function assay showed that administration of *P. gingivalis* increased gut permeability compared with the sham group (**Figure 5B**). Moreover, we determined the expression of junctional proteins located in the gut. As the results indicated, administration of *P. gingivalis* significantly decreased the expression of ZO-1, a major component of tight junction proteins (**Figures 5C, D**), and E-cadherin, a major component of adhesion molecules (**Figures 5E, F**).

### Serum Metabolic Profile and Analysis With *P. gingivalis* Administration

**Figure 6:**
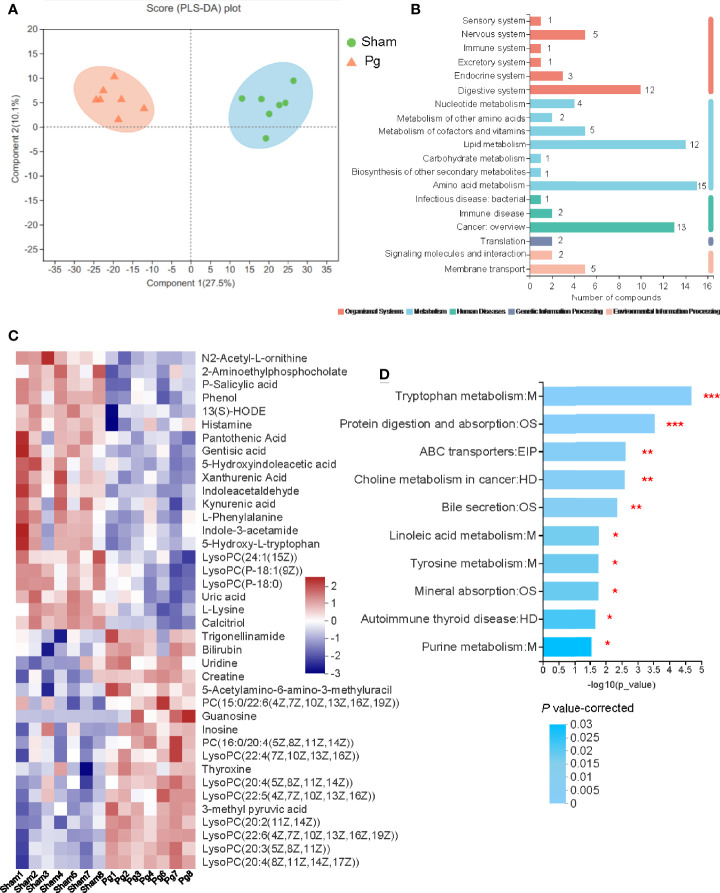
Untargeted metabolomics of the serum metabolomes with *P. gingivalis* administration. **(A)** Principal components analysis (PCA) scores of serum metabolites in mice between the sham and *Pg* groups (n = 7 per group). **(B)** Pathways of all the serum metabolites distributed in mice between the sham and *Pg* groups. **(C)** Heatmap of the 39 significantly expressed metabolites in metabolism related pathways between sham and *Pg* groups. **(D)** 10 of the top enriched KEGG pathways related to metabolism in the sham and *Pg* groups, and the *P* value was corrected with Benjamini and Hochberg method. *P < 0.05; **P < 0.01; ***P < 0.001.

Microbial metabolites are key actors in host-microbiota crosstalk, and we further analyzed the serum samples of *P. gingivalis*- and sham-administered mice with untargeted metabolomics. The principal component analysis (PCA) based on nuclear magnetic resonance demonstrated a divergent separation of the serum metabolic profile between *P. gingivalis*- and sham-administered mice (**Figure 6A**). A total of 568 metabolites were identified in both groups, and 80 metabolites were upregulated while 76 metabolites were downregulated according to a variable value set at a VIP > 1 and a *P* value < 0.05 in the Wilcoxon rank-sum test (**Table S1**). The pathway classification showed a total of 88 metabolites enriched in metabolic pathways, including lipid metabolism and amino acid metabolism (**Figure 6B**). The heatmap analysis identified 39 metabolites related with metabolic pathways, with 18 metabolites that were upregulated and 21 metabolites that were downregulated with *P. gingivalis* administration (**Figure 6C**). The analysis of the metabolic pathways demonstrated 10 of the most relevant pathways associated with the metabolites in the *Pg* and sham groups, including tryptophan metabolism, protein digestion and absorption, and choline metabolism (**Figure 6D**).

### Correlation Analysis Between Gut Microbiota and Serum Metabolome

**Figure 7:**
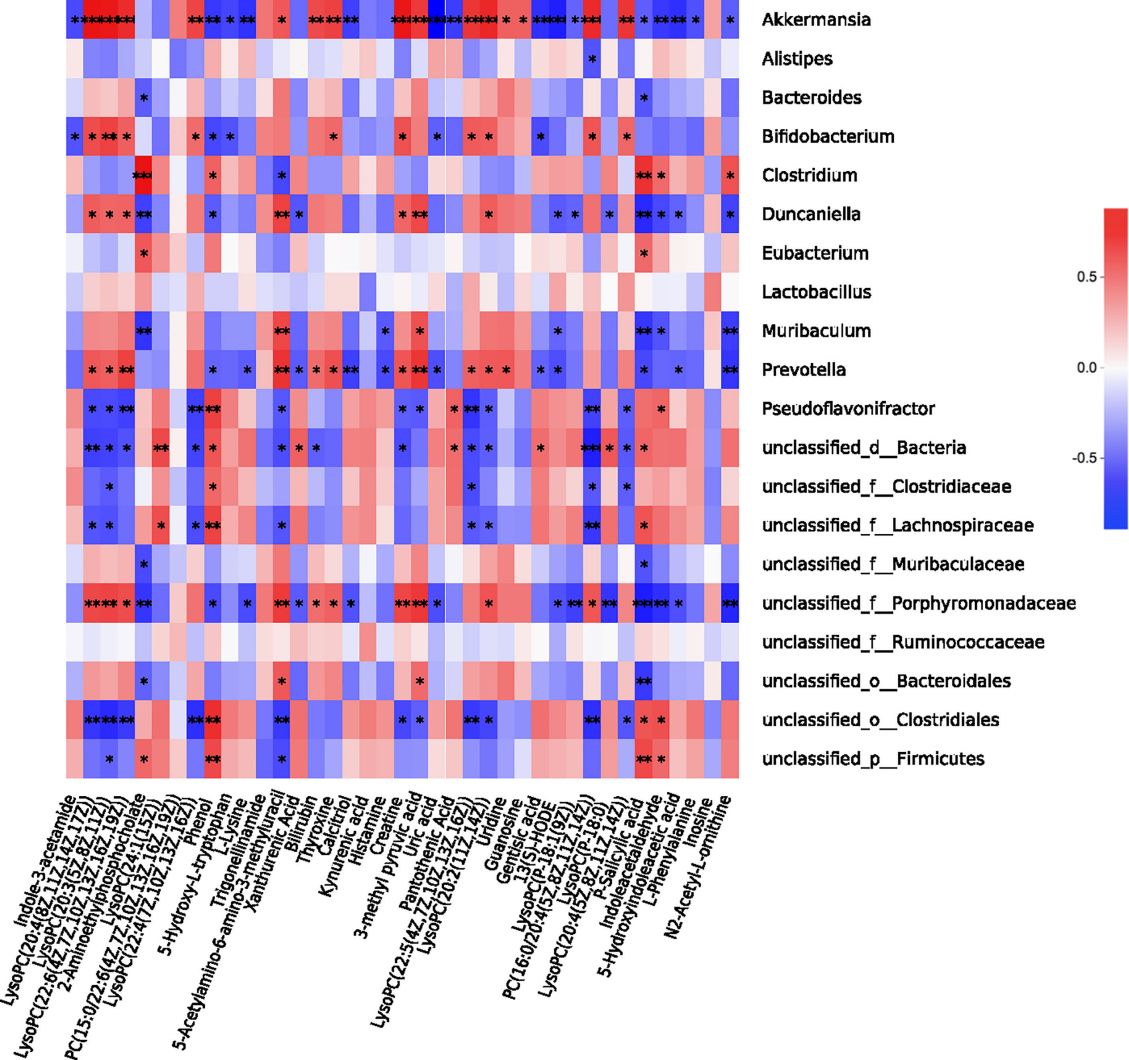
Correlation analysis between gut microbiota and serum metabolome. Correlation heatmap of the 39 most dominant serum metabolites and intestinal genera between sham and *Pg* groups. *P < 0.05; **P < 0.01; ***P < 0.001; ****P < 0.0001.

The above results demonstrated that *P. gingivalis* administration induces metabolic disorders, gut microbiota dysbiosis and serum metabolome alterations. As gut microbiota interact with the host to produce metabolites, acting as intermediates or end-products of microbial metabolism, we further performed a correlation analysis involving the above 39 metabolites with all of the bacterial genera (**Figure 7**). *Porphyromonadaceae*, to which *P. gingivalis* belongs, showed a wide correlation with most metabolites, such as 5-hydroxyindoleacetic acid, indole-3-acetaldehyde (IAAld), P-salicylic acid, phosphatiylcholine (PC), and creatine (r = 0.61, 0.72, 0.79, and 0.69, respectively, **Table S2**). *Akkermansia* has been shown to be correlated with almost all of the metabolites. PC, as the precursor of trimethylamine (TMA) and TMAO, has been demonstrated to be positively correlated with *Prevotella* and *Porphyromonadace* and negatively correlated with *Lachnospiraceae* and *Clostridiales*. These results indicate that serum metabolome alteration and gut microbiota dysbiosis are closely related to *P. gingivalis* administration.

## Discussion

MetS represents a series of metabolic disorders with obesity-induced proinflammatory states as the putative cause, in which adipocytes release proinflammatory factors and recruit macrophages to accelerate systemic inflammation during the development of MetS (Winer et al., 2016). Sharing some inflammatory effector mechanisms, recent studies have focused on the effect of periodontitis on MetS, and epidemiological research has indicated that the prevalence of MetS is significantly increased in patients with periodontitis compared with those without periodontitis (Kim et al., 2018). As a biofilm-triggered disease, periodontitis is characterized by progressive destruction of tooth-supporting tissues and dysbiosis of the oral microbiota (Lamont et al., 2018). The latter plays a vital role in linking periodontal disease with systematic disorders. Studies have shown that up to 10^12^ free bacteria per day are swallowed by patients with periodontitis (Boutaga et al., 2007; Saygun et al., 2011). In the present study, HFD-induced and *P. gingivalis*-administered mice exhibited similar metabolic disorders, including adipocyte hypertrophy, macrophage infiltration, intestinal barrier defects, and insulin resistance. Unlike HFD, however, *P. gingivalis*-administered mice showed an increase in the proportion of body fat rather than body weight.

The prevailing viewpoints suggest that intestinal dysbiosis and barrier defects triggered by a high-fat and low-fiber diet facilitate the passage of bacterial metabolites into circulation. Some studies have demonstrated alterations in the gut microbiota in mice with *P. gingivalis* administration. For example, Tsuzuno et al. detected elevated proportions of unclassed *Coriobacteriaceae*, *Gemellaceae*, and *Clostridiaceae* and decreased proportions of *Prevotellaceae*, *Butyricicoccus*, and *Bilophila* in *P. gingivalis*-administered mice *via* 16S rRNA sequencing (Tsuzuno et al., 2021). The composition and abundance of gut microbiota was not exactly the same as our results, but both studies showed similar separation tendencies between *P. gingivalis*-administered mice and the sham group. In our study, the proportion of unclassified *Lachnospiraceae* was decreased in *P. gingivalis*-administered mice, while the proportions of unclassified *Muribaculaceae*, *Akkermansia*, *Prevotella* and *Porphyromonadaceae* were increased. As one of the most abundant members of the gut microbiota, *Akkermansia* mainly resides in the gut mucus layer to restore or increase the thickness of the mucus layer. Therefore, accumulating studies have indicated *Akkermansia* as a potential probiotic (Cani and de Vos, 2017; Kequan, 2017; Kim et al., 2020). However, some research has indicated that *Akkermansia* might play a negative role. A metagenome-wide association study based on 345 Chinese T2D patients and nondiabetic controls demonstrated that the abundance of *Akkermansia* was positively correlated with T2D disorders (Qin et al., 2012). Moreover, in certain circumstances, for example, *IL-10* knockout mice, *Akkermansia* could promote colitis and MetS by destroying the mucus layer (Chassaing et al., 2015). Here, we proposed that *Akkermansia* is not a probiotic but rather a mucus consumer that destroys the mucus layer, leading to increased gut permeability and metabolic disorders.

Moreover, a decreased *Firmicutes*/*Bacteroidetes* (F/B) ratio was detected with *P. gingivalis* administration in our study, which is contrary to some research. As *Firmicutes* and *Bacteroidetes* are predominant microbial phyla in gut microbiota, previous studies often supported an increased F/B ratio as a relevant event of metabolic diseases (Ley et al., 2006). However, some contrary results make this claim controversial. Ley et al. (Carvalho et al., 2012). found *via* metagenomic analysis that the F/B ratio in HFD mice was downregulated and elevated along with improved metabolic indicators after antibiotic treatment. Moreover, Schwiertz et al. exhibited a significantly upregulated proportion of *Bacteroidetes* in patients with obesity, leading to a decreased F/B ratio (Schwiertz et al., 2010). With our deepening understanding of the gut microbiota and the development of combined sequencing, the role and effect of a specific microbiome and its metabolite products have been shown to be key factors in the pathogenesis of MetS.

Here, untargeted metabolomics was introduced in our study to investigate the whole spectra of metabolites in *P. gingivalis*-administered mice. According to the results, a total of 568 metabolites were characterized in the two groups, of which 80 were upregulated and 76 were downregulated with *P. gingivalis* administration. Most of the differential metabolites were involved in metabolic pathways, including tryptophan metabolism, protein digestion and absorption, choline metabolism and bile secretion. Among them, tryptophan metabolism and the choline pathway are of particular focus.

As tryptophan is an essential aromatic amino acid, tryptophan metabolism involves three main metabolic pathways in the intestinal tract, and its products include ligands of the aryl hydrocarbon receptor (AhR), kynurenic acid, and 5-hydroxytryptamine (5-HT) (Zelante et al., 2013; Yano et al., 2015; Kennedy et al., 2017; Agus et al., 2018). Notably, most of the metabolites, such as IAAld, indole-3-acetamide (IAM), and 5-hydroxyindoleacetic acid, were reduced in the *P. gingivalis*-administered mice, indicating that a deficiency of tryptophan metabolism might be a relevant event in the pathogenesis of *P. gingivalis*-associated MetS. It has been well established that AhR signaling plays an important role in maintaining intestinal barrier function, such as intestinal immune balance, resistance to pathogen invasion, and epithelial cell renewal (Lamas et al., 2018). Here, along with the reduction in indole metabolites, intestinal barrier function was also destroyed with *P. gingivalis* administration, characterized by decreased expression of the tight junction protein ZO-1 and the adhesion molecule E-cadherin. Until now, only two bacterial microbiota have been elucidated to produce tryptophan (Perdew et al., 2015), one of which is *Lactobacillus*, whose abundance was significantly reduced with *P. gingivalis* administration. Through aromatic amino acid aminotransferase (ArAT) and indole-lactate dehydrogenase (ILDH), *Lactobacillus* can convert tryptophan to indole-3-aldehyde (IAld) and indole-3-lactic acid (ILA) (Roager and Licht, 2018). Thus, the intestinal barrier function of lactobacillius indole and its derivatives are linked, which might be a pathogenic pathway underlying oral pathogen-induced MetS.

In addition to tryptophan metabolism, a series of choline-related metabolites have also been characterized as differentially elevated metabolites with *P. gingivalis* administration. Of note, choline, betanie and L-carnitine are important intermediates that produce TMA and TMAO, which have been implicated in the progression of cardiovascular diseases, T2D, insulin resistance, nonalcoholic fatty liver disease, and certain cancers (Jens et al., 2017; Tang and Hazen, 2017; Tan et al., 2019). A variety of intestinal bacteria have been found to metabolize choline into TMA and TMAO. A recent study indicated that as the proportion of *Prevotella* increased in the intestinal flora, the content of serum TMA/TMAO was significantly increased (Koeth et al., 2013), which was also identified according to our results.

In conclusion, our study demonstrated that oral administration of *P. gingivalis* could induce dysbiosis of the gut microbiota and its derived metabolites with the development of metabolic disorders and destruction of intestinal barrier function. These findings provide novel insights into potential links between periodontal disease and MetS. Nevertheless, administration of a single species of periodontopathic bacteria does not completely replicate the conditions of periodontitis patients. Moreover, although we have screened some metabolites such as indole and its derivatives in the pathogenesis of metabolic disorders arose by *P. gingivalis*, the specific mechanism is not certain. Further studies are needed to investigate the precise mechanism in the pathogenesis of periodontitis-associated MetS.

## Data Availability Statement

The datasets presented in this study can be found in online repositories. Metagenomic sequencing data have been deposited in NCBI sequencing read archive (SRA) with the accession number SRP362079. Untargeted metabolomics data have been deposited in MetaboLights with the accession number MTBLS4106.

## Ethics Statement

The animal study was reviewed and approved by the Animal Experimental Ethics Committee of Fudan University (No. 202202006S).

## Author Contributions

ZD and SC designed the study. ZD and CZ conducted experiments. WL analyzed the data. CZ and SC prepared and revised the manuscript. All the authors contributed to the article and approved the submitted version.

## Funding

This study was financially supported by grants from National Natural Science Foundation of China (82001056), and Key Laboratory of Fudan University Molecular Engineering of Polymers (2021-06).

## Conflict of Interest

The authors declare that the research was conducted in the absence of any commercial or financial relationships that could be construed as a potential conflict of interest.

## Publisher’s Note

All claims expressed in this article are solely those of the authors and do not necessarily represent those of their affiliated organizations, or those of the publisher, the editors and the reviewers. Any product that may be evaluated in this article, or claim that may be made by its manufacturer, is not guaranteed or endorsed by the publisher.
